# Preface

**DOI:** 10.1093/jxb/erv395

**Published:** 2015-08-19

**Authors:** Roland Pieruschka, Tracy Lawson

**Affiliations:** Institute for Bio- and Geosciences, Jülich, Germany; University of Essex, UK

One of the current challenges in plant biology is the development of quantitative phenotyping approaches to link the genotype and the environment to plant structural, functional, and yield characteristics in order to meet the growing demands for sustainable food, feed, and fuel. The genotype of a plant consists of all of the hereditary information within the individual, whilst the phenotype, which represents the morphological, physiological, anatomical, and developmental characteristics, is the result of the interaction between the genotype and the environment. Understanding this interaction is one of the major challenges in plant sciences. In plant breeding, the ultimate goal is the improvement of traits of agricultural importance related to disease resistance, high yields, and the plant’s ability to grow in unfavourable environmental conditions. Currently, breeding approaches produce an annual yield increase of approximately 1% for major crops, which is below the over 2% increase needed to meet the global demands for food by 2050 (Ray *et al.*, 2013).

Rapid developments in plant molecular biology and in molecular-based breeding techniques have resulted in an increasing number of species being sequenced and large collections of mutants, accessions, and recombinant lines allowing detailed analysis of gene functions. High-definition genotyping can now be carried out on thousands of plants in an automated way at continuously decreasing costs, thereby facilitating association genetics and the determination of multi-parental quantitative trait loci (QTLs) (Poland and Rife, 2012). For transcriptomic, proteomic, and metabolomic analyses large, often robotized, platforms are available allowing detailed characterization of the biochemical status of plants at a reasonable cost (Ehrhardt and Frommer, 2012). By contrast, an understanding of the link between genotype and phenotype has progressed more slowly and is the major limiting step in current breeding programmes to enable further increases in plant productivity. Faster progress requires a substantial increase in capacity (both technical and conceptual) of the plant science community to analyse quantitatively the existing genetic resources for their interaction with the environment. Advances in phenotyping are, therefore, the key factor for success in modern breeding, as well as for basic plant research, and this requires a multidisciplinary effort which includes the integration of sensor networks, environment simulation facilities, specialized platforms for mechanistic, medium- and high-throughput platforms, field phenotyping systems, and modelling (Fiorani and Schurr, 2013).

Despite the fact that our understanding about the link between the genotype and the phenotype is advancing and new species and traits can be addressed, we will never be able experimentally to explore the entire genotype–environment matrix for individual factors and their interactions (see, for example, Mittler and Blumwald, 2010). Therefore, modelling platforms are needed to test the existing or virtual combinations of alleles in a variety of climatic scenarios or management practices to make relevant predictions about phenotypes. This requires collection, storage, and access to phenotypic data, based on standards which allow interoperability among data providers, and to link specific phenotypes with the associated genomic sequence information. This is particularly challenging because of the large variability of phenotyping protocols, the multitude of phenotypic traits which can be measured, and their dependence on the environment. Establishing standardized procedures such as experimental protocols and environmental monitoring, as well as data managements that allow comparability between measurements, is the next important challenge which requires an effort beyond the plant science community and extended interaction between different stakeholders.

In addition, close co-operation and interaction between scientists establishing plant phenotyping platforms, technology developers, as well as other diverse users, is of great importance to foster a discussion about the needs and requirements of plant phenotyping. There is a number of national and international networks (e.g. APPF, http://www.plantphenomics.org.au/; DPPN, www.dppn.de; FPPN, https://www.phenome-fppn.fr/; UK-PPN, http://www.ukppn.org.uk/) as well as projects (e.g. EPPN, http://www.plant-phenotyping-network.eu/; COST action, http://costfa1306.eu/) or initiatives focusing on specific crops (G20, http://www.wheatinitiative.org/activities/expert-working-groups), which aim to foster the discussion between the various stakeholders. In particular, the International Plant Phenotyping Network (IPPN, http://www.plant-phenotyping.org/) focuses on wide international interaction by organizing bi-annual symposia and promoting discussion and interaction between scientists and industry (http://www.plant-phenotyping.org/previous_events). The first two symposia in Canberra in 2009 and Jülich in 2011 had a strong focus on encouraging the development and spread of new techniques and algorithms for the automation of analyses as well as data management frameworks enabling modelling as depicted in the resulting special issues (Furbank, 2009; Pieruschka and Poorter, 2012). The latest symposium in Chennai in 2014 indicated that plant phenotyping is becoming a tool to be used to address complex traits such as yield, plant biomass, and plant productivity in response to biotic and abiotic stress. Thus, plant phenotyping as a research field is developing towards phenotyping for the diverse needs of users, which is accompanied by the development and implementation of novel technology to effectively address the phenotyping bottleneck. This special issue on plant phenotyping initiated by the Symposium in Chennai underlines this development with publications about phenotyping for biotic and abiotic stress resistance of different crops accompanied by a descriptions of methods, quantitative approaches, and platforms for phenotyping of specific traits.

## Content of this special issue

Phenotyping root traits is of particular importance for improved uptake of resources such as water and nutrients. Kuijken *et al.* (2015) discuss the heritability of root traits in relation to relevant breeding targets. Niederbacher *et al.* (2015) present an approach to define disease resistance and stress tolerance by measuring volatile organic compounds. Detailed physiological phenotyping across different scales is proposed to integrate the precise characterization of metabolic processes into high-throughput phenotyping of whole-plants and canopies (Großkinsky *et al.*, 2015). The importance of standardization is discussed by Krajewski *et al.* (2015) who highlight the problems and shortcomings as well as the benefits. A number of contributions deal with plant properties under different stress conditions. Soil moisture affects root and shoot properties, in particular, under different irradiances that plants are exposed to (Nagel *et al.*, 2015). Water availability was studied with respect to different lentil genotypes in combination with salinity stress indicating different biochemical markers (Muscolo *et al.*, 2015); remote sensing approaches used to screen apple populations revealed different QTLs (Virlet et al 2015); and Parent *et al.* (2015) showed a possible common genetic basis linking greenhouse and field measurements. York and Lynch (2015) found different root architecture under varying nitrogen availability which affects plant growth. The effect of domestication on root and shoot properties under different nutrient availability was investigated by Gioia *et al.* (2015) showing a significantly reduced diversity; In ‘t Zandt *et al*. (2015) designed different nutrient supply treatments using a rhizoslide system. Jammer *et al.* (2015) studied the key carbohydrate metabolic enzyme dynamics of crops exposed to different environmental conditions such as elevated CO_2_ or heat stress. Infection of sugar beets with *Cercospora* significantly affects the growth and morphological dynamics of taproots (Schmittgen *et al.*, 2015). Genetic components of early vigour in rice (Rebolledo *et al.*, 2015) or growth dynamics in *Arabidopsis* (Bac-Molenaar *et al.*, 2015) were studied with dedicated mapping populations. In addition, different methods and approaches are discussed, such as methods for phenotyping photosynthetic performance using the sun-induced fluorescence approach (Tubuxin *et al.*, 2015) or evaluation of vegetation indices for remote chlorophyll measurement when measured on ad- and abaxial surfaces of poplar leaves (Lu *et al.*, 2015). Yang *et al.* (2015) presents a leaf scorer which is implemented into a phenotyping platform and validated in a genome-wide association study in rice. A new platform for 3-D imaging and lysimetry was specifically developed for the phenotyping of drought stress by Vadez *et al.* (2015). Hatzig *et al.* (2015) use a neural network approach to quantify root architecture which was tested under drought stress. In summary, this special issue deals with very diverse topics relevant for plant phenotyping such as an understanding of basic plant processes, the identification of genetic regions related to different physiological processes, the development and implementation of novel methods to identify novel traits etc. Thus, within the last few years, plant phenotyping has become a more and more important tool to quantify the link between the genotype and the environment that is highly relevant to address future grand challenges.
